# Supplementary material for: Left hemisphere dominance for bilateral kinematic encoding in the human brain
Source: eLife. 2022 Mar 1;11:e69977. doi: 10.7554/eLife.69977 (PMC8887902; doi:10.7554/eLife.69977)
Supplement: Transparent reporting form [file elife-69977-transrepform1.pdf]

eLife

Westbrook Centre
Milton Road
Cambridge CB4 1YG, UK

P 01223 855340
W elifesciences.org
T @elife

# *eLife's* transparent reporting form

We encourage authors to provide detailed information *within their submission* to facilitate the interpretation and replication of experiments. Authors can upload supporting documentation to indicate the use of appropriate reporting guidelines for health-related research (see EQUATOR Network), life science research (see the BioSharing Information Resource), or the ARRIVE guidelines for reporting work involving animal research. Where applicable, authors should refer to any relevant reporting standards documents in this form.

If you have any questions, please consult our Journal Policies and/or contact us: editorial@elifesciences.org.

**Sample-size estimation**

- You should state whether an appropriate sample size was computed when the study was being designed
- You should state the statistical method of sample size computation and any required assumptions
- If no explicit power analysis was used, you should describe how you decided what sample (replicate) size (number) to use

Please outline where this information can be found within the submission (e.g., sections or figure legends), or explain why this information doesn't apply to your submission:

> Although it is often difficult to obtain large numbers of patients for intracranial datasets, in our paper we have clean neural data from over 660 ECoG electrodes across 6 patients. No explicit power analyses were used, in part because we do not rely on classical statistics to examine our neural effects. In our paper, each electrode was fit individually with a kinematic encoding model and performance was assessed on held-out trials ensuring that we did not overfit the model to the data. Based on the held-out performance we then used non-parametric permutation tests to examine differences between hemispheres and task phases. Thus, although we do not have a large patient sample, we focus on analyses that reduce overfitting and avoid assumptions of parametric tests.

**Replicates**

- You should report how often each experiment was performed
- You should include a definition of biological versus technical replication
- The data obtained should be provided and sufficient information should be provided to indicate the number of independent biological and/or technical replicates
- If you encountered any outliers, you should describe how these were handled
- Criteria for exclusion/inclusion of data should be clearly stated
- High-throughput sequence data should be uploaded before submission, with a private link for reviewers provided (these are available from both GEO and ArrayExpress)

Please outline where this information can be found within the submission (e.g., sections or figure legends), or explain why this information doesn't apply to your submission:

> Detailed explanation of how the experiment was performed are included in the method section along with the exact procedures that were used in order to pre-process the neural data. Inclusion and exclusion criteria for electrodes are clearly listed.

eLife Sciences Publications, Ltd is a limited liability non-profit non-stock corporation incorporated in the State of Delaware, USA, with company number 5030732, and is registered in the UK with company number FC030576 and branch number BR015634 at the address Westbrook Centre, Milton Road, Cambridge CB4 1YG.

**Statistical reporting**

- Statistical analysis methods should be described and justified
- Raw data should be presented in figures whenever informative to do so (typically when N per group is less than 10)
- For each experiment, you should identify the statistical tests used, exact values of N, definitions of center, methods of multiple test correction, and dispersion and precision measures (e.g., mean, median, SD, SEM, confidence intervals; and, for the major substantive results, a measure of effect size (e.g., Pearson's r, Cohen's d)
- Report exact p-values wherever possible alongside the summary statistics and 95% confidence intervals. These should be reported for all key questions and not only when the p-value is less than 0.05.

Please outline where this information can be found within the submission (e.g., sections or figure legends), or explain why this information doesn't apply to your submission:

Statistical analysis methods are outlined in Results whenever test results are cited and described in detail in the Method section under – Permutation-based linear mixed effects model. The full distribution of electrode prediction performances from the encoding model are included in the figures to show central tendencies as well as the spread of the data. All advanced metrics are described in Methods with thorough mathematical formulation. Exact p-values are not given because permutation tests can result in different *p*-values.

datasets, or papers with a very large number of statistical tests, you may upload a single table file with tests, Ns, etc., with reference to sections in the manuscript.)

**Group allocation**

- Indicate how samples were allocated into experimental groups (in the case of clinical studies, please specify allocation to treatment method); if randomization was used, please also state if restricted randomization was applied
- Indicate if masking was used during group allocation, data collection and/or data analysis

Please outline where this information can be found within the submission (e.g., sections or figure legends), or explain why this information doesn't apply to your submission:

In our paper we compare between recording hemisphere which was based on a clinical assessment for each patient that was independent of research. Patient was used as a random factor in all our linear mixed effect models. All patients completed 2 blocks of reaches with either the arm contralateral or ipsilateral to their subdural

**Additional data files ("source data")**

- We encourage you to upload relevant additional data files, such as numerical data that are represented as a graph in a figure, or as a summary table
- Where provided, these should be in the most useful format, and they can be uploaded as "Source data" files linked to a main figure or table
- Include model definition files including the full list of parameters used
- Include code used for data analysis (e.g., R, MatLab)
- Avoid stating that data files are "available upon request"

Please indicate the figures or tables for which source data files have been provided:

eLife Sciences Publications, Ltd is a limited liability non-profit non-stock corporation incorporated in the State of Delaware, USA, with company number 5030732, and is registered in the UK with company number FC030576 and branch number BR015634 at the address Westbrook Centre, Milton Road, Cambridge CB4 1YG.

All data used in the manuscript have been uploaded to Zenodo and have been made publicly available: https://zenodo.org/record/4761390#.YJ8vPJNKh24. Code to run patients through the kinematic encoding model are available on git at: https://github.com/cmerrick15/Asymmetry_ECoG_dataset

eLife Sciences Publications, Ltd is a limited liability non-profit non-stock corporation incorporated in the State of Delaware, USA, with company number 5030732, and is registered in the UK with company number FC030576 and branch number BR015634 at the address Westbrook Centre, Milton Road, Cambridge CB4 1YG.
